# Understanding Uptake of Digital Health Products: Methodology Tutorial for a Discrete Choice Experiment Using the Bayesian Efficient Design

**DOI:** 10.2196/32365

**Published:** 2021-10-11

**Authors:** Dorothy Szinay, Rory Cameron, Felix Naughton, Jennifer A Whitty, Jamie Brown, Andy Jones

**Affiliations:** 1 Behavioural and Implementation Science Group School of Health Sciences University of East Anglia Norwich United Kingdom; 2 Norwich Medical School University of East Anglia Norwich United Kingdom; 3 National Institute for Health Research Applied Research Collaboration East of England Cambridge United Kingdom; 4 Department of Behavioural Science and Health University College London London United Kingdom; 5 SPECTRUM Consortium London United Kingdom

**Keywords:** discrete choice experiment, stated preference methods, mHealth, digital health, quantitative methodology, uptake, engagement, methodology, preference, Bayesian, design, tutorial, qualitative, user preference

## Abstract

Understanding the preferences of potential users of digital health products is beneficial for digital health policy and planning. Stated preference methods could help elicit individuals’ preferences in the absence of observational data. A discrete choice experiment (DCE) is a commonly used stated preference method—a quantitative methodology that argues that individuals make trade-offs when engaging in a decision by choosing an alternative of a product or a service that offers the greatest utility, or benefit. This methodology is widely used in health economics in situations in which revealed preferences are difficult to collect but is much less used in the field of digital health. This paper outlines the stages involved in developing a DCE. As a case study, it uses the application of a DCE to reveal preferences in targeting the uptake of smoking cessation apps. It describes the establishment of attributes, the construction of choice tasks of 2 or more alternatives, and the development of the experimental design. This tutorial offers a guide for researchers with no prior knowledge of this research technique.

## Introduction

Understanding how the public values different aspects of digital health tools, such as smoking cessation or physical activity apps, can help providers of the tools to identify functionality that is important to users, which may improve uptake (ie, selection, download, and installation of apps) [1]. This is important because uptake of digital tools is generally low. More information regarding the preferences of users when selecting a digital health tool, for example via an app store, may allow providers to present their products in such a way that may increase their uptake. However, pragmatic challenges, such as examining how each potentially modifiable aspect of a digital health product (eg, presentation, design, and features that it offers) or intervention design will impact preference or the choice of uptake, often mean this is not feasible or practical [2]. Therefore, increasing attention is being paid toward stated preference methods to understand preferences when designing digital health products and services, with examples including COVID-tracing apps [3,4], sun protection apps to prevent skin cancer [5], and the uptake of health apps in general [6].

Stated preference methods are survey-based methods aiming to elicit individuals’ preferences toward a specific behavior, particularly those that are not well understood. The most widely used type of stated preference method is the discrete choice experiment (DCE) [7]. According to Spinks et al [8], Louviere and Hensher (1982) and Louviere and Woodworth (1983) originally developed DCEs to study the marketing and economics of transport, and the fields of psychology and economics have profoundly influenced the DCE methodology since it was developed. In recent years, DCEs have been increasingly used in health and health care settings [9,10], as well as in addiction research [11] and digital health [4-6]. The increasing number of DCEs in digital health highlights their potential, although they are currently underused.

Discrete choice differentiates from other stated preference methods in the way that responses are elicited [12]. The DCE uses a survey-based experimental design, where participants are presented with a series of hypothetical scenarios. In these scenarios, participants are shown situations, known as *choice tasks*. Attempting to mimic real-world decision making, in each choice task, participants then have to choose a product or a service from two or more options, known as *alternatives* [13]. Each alternative consists of a set of characteristics, known as *attributes*, with at least two types, known as *attribute levels* [13]. Participants are asked to choose a preferred alternative in each choice task, which allows researchers to quantify the relative strength of preferences for improvements in certain attributes [8,14].

The outputs from statistical models developed using DCE data can be beneficial for estimating uptake of new products or services, including digital health tools, where observational data are not available or are difficult to obtain otherwise [15,16]. Lack of observational data often implies a requirement to seek scientific views and comments from experts in order to generate predictions of a target behavior [17]. However, DCEs can provide an empirical alternative to expert opinions, while accounting for possible interactions between attributes (eg, design of a product and brand name), which are otherwise often ignored [18].

In our research, we wanted to understand how to present health apps on curated health app portals to increase their uptake. This paper describes the development of a DCE in digital health that aims to elicit potential user preferences on smoking cessation app uptake. It explains how the attributes and their levels are selected and describes the construction of choice tasks and the experimental design. The study protocol of the research this paper is based on is registered on the Open Science Framework [19].

## Development of a DCE

The development of a DCE should follow published recommendations, including the checklist for good research practices [9], guides on the development of a DCE [13,20], recommendations on how to construct the experimental design [7,20-23], and which statistical methods can be used [24].

### Establishing Attributes

An important step in designing a DCE is the identification of the relevant attributes for the subject matter. Attributes in a DCE can be quantitative, such as cost, or qualitative, such as the design of a product [25]. The identification of attributes is typically based on primary and secondary data collection to ensure that the DCE is tailored to the study setting [13]. It should ideally commence with a literature review that will inform qualitative research to identify relevant attributes [26]. Although there is no set limit on the number of attributes that can be included in a DCE, to ensure that the cognitive load of the participants is manageable, it should be less than 10 [13], with a general expectation to include 5-7 attributes [27].

Our DCE was based on a comprehensive systematic review investigating factors influencing the uptake and engagement with health and well-being smartphone apps [28] and a qualitative research component that consisted of a think-aloud and interview study to examine further the previously identified factors or attributes [29]. The importance of qualitative research lies in ensuring inclusion of attributes that are relevant to most participants [25]. Of the 14 factors initially identified as being relevant for the uptake of health and well-being apps, 5 were retained and included in the DCE: the monthly price of the app, who developed the app, the star ratings of the app, the description of the app, and images shown. These factors were chosen due to their perceived importance during our previous qualitative research and for pragmatic reasons, including how easily measurable and presentable they were within the DCE.

An important step in designing a DCE is in ensuring the content validity of the instrument: the identification of relevant attributes for the subject matter. Following administration of the survey, methods are available for the measurement and assessment of the content validity of the instrument, although their use is not widely reported [30].

#### Establishing Attribute Levels

The next step is to establish attribute levels. The level of an attribute must also be of a range that ensures a trade-off between attributes. A trade-off is defined as an exchange in which a participant gives up some amount of one attribute to gain more of another. It has been suggested that increasing the number of levels for an attribute increases the relative importance of that attribute [31] and that imbalance in the numbers of levels across attributes raises the importance of the attributes with higher levels [32]. Yang et al [32] suggested that a balance exists between simpler designs with lower numbers of levels, which reduce the respondent burden (and consequently measurement error) and are useful for identifying attribute rankings, and more complex designs with higher levels (and higher statistical precision) and is more sensitive to identifying trade-offs between attributes. Based on this, and the commonly adopted practices in the research field, we aimed to include at least three levels for each attribute.

**Figure 1:**
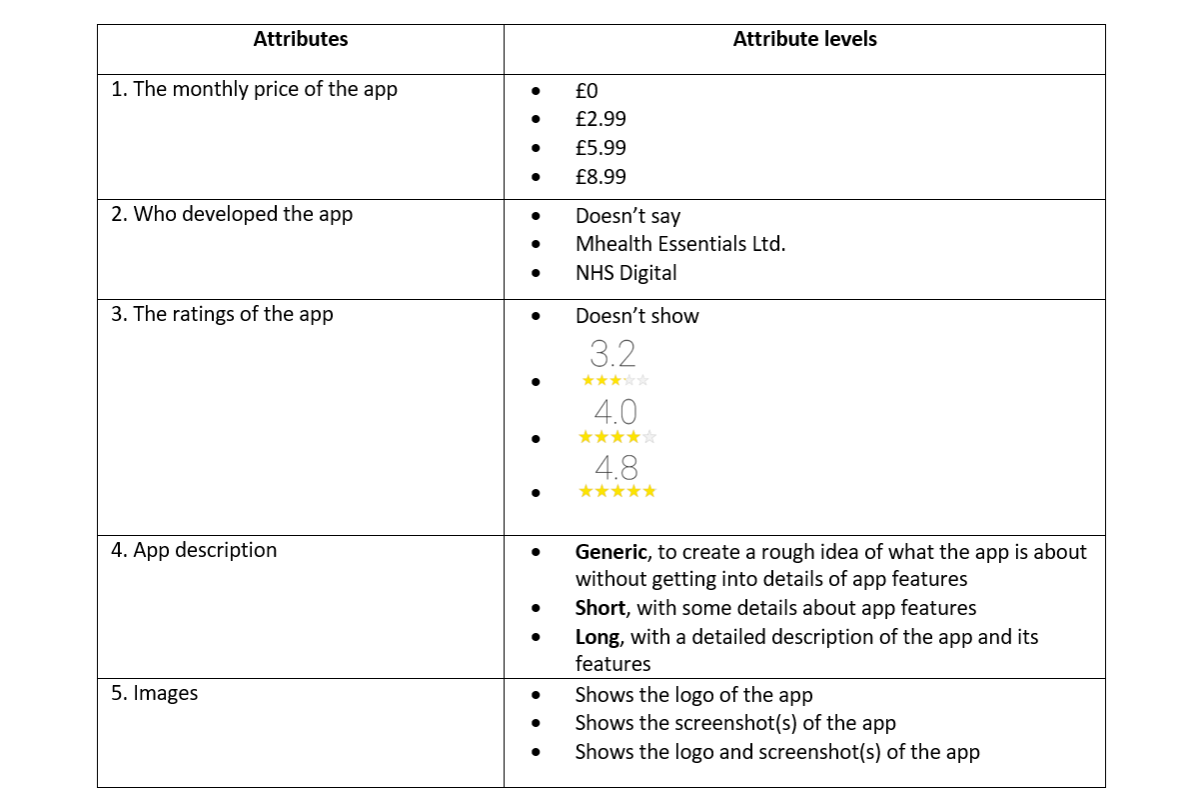
Attributes and attribute levels in our DCE. DCE: discrete choice experiment.

If a range is not suitable, participants might consider the differences between levels unimportant [25]. For example, the difference between the star ratings of 4.8 and 4.7 for a smoking cessation app is not as relevant as the difference between 4.8 and 4. In our research, to refine attribute levels, a survey was conducted with 34 participants. In the survey, the levels of two attributes we were unsure of (the monthly price of the app and the ratings) were carefully considered in order to specify at a sufficiently wide range so that the difference between the levels would likely make a difference in response. When a range is not wide enough, there is a risk that participants could ignore the attributes because they judge the difference between levels to be insignificant [20]. See Figure 1 for the final list of attributes and levels included in our DCE.

### Choice Tasks

Once the attributes and their levels are identified, the decision to develop full- or partial-profile tasks with or without an opt-out option needs to be made. A full profile refers to the display of all five attributes in both alternatives in each choice set. A partial profile DCE will not present certain attributes for certain alternatives. For example, if a DCE is used to investigate the trade-off between a higher number of attributes (eg, a total of nine attributes), it could be beneficial to limit the number of attributes shown at one time (eg, five attributes) to limit participant cognitive load. Five attributes are generally considered low enough to complete a full-profile choice task, which consequently maximizes the information about trade-offs [33]. Hence, in our research, we applied a full-profile DCE.

**Figure 2:**
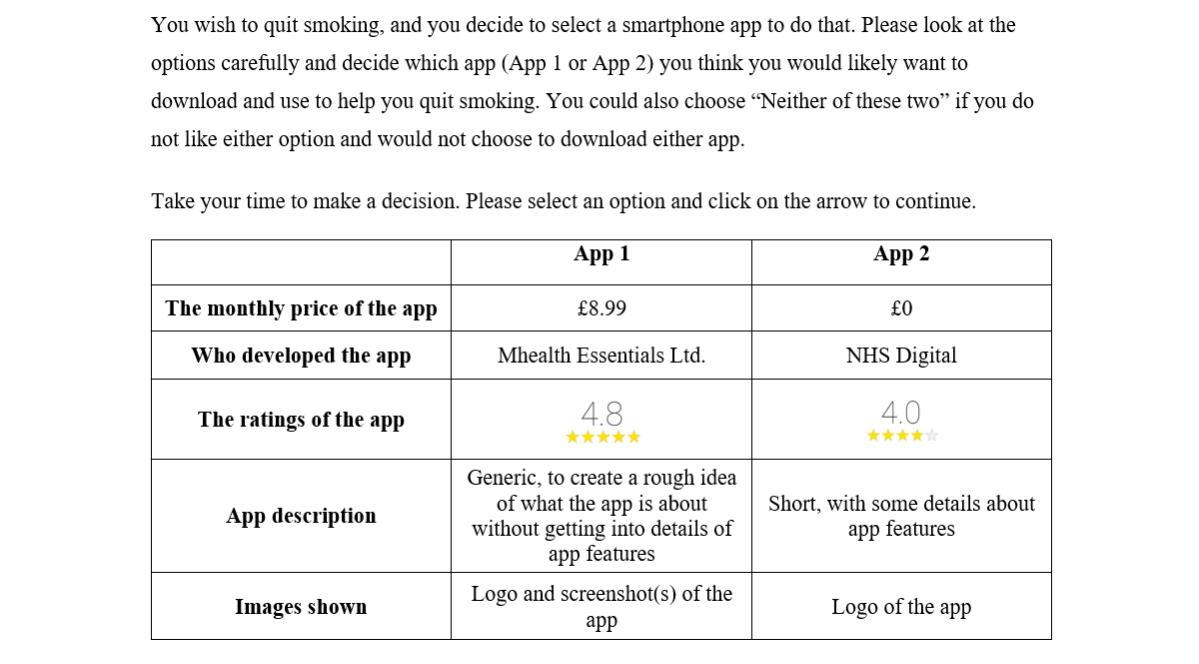
An example of a scenario with an opt-out option used in our DCE. DCE: discrete choice experiment.

A neutral option (“Neither of these 2”), known as an opt-out alternative, was included, in addition to selecting alternative apps. The opt-out option has the potential to make the choices more realistic [34] by simulating a real-world context where individuals can exercise their right not to take up an app, given the apps on offer [20]. In our DCE, a participant had the option to choose or reject the hypothetical uptake of a smoking cessation app. However, when a participant selects the opt-out option, no information is provided on how they trade-off attribute levels or alternatives [13]. In some situations, a *forced-choice* scenario can be included, where participants who chose the opt-out option are prompted to make a choice regardless. An example of a scenario with an opt-out option is shown in Figure 2.

### Experimental Design

An experimental design is a systematic method of generating choice sets that are presented to respondents. This enables the specification of the choice sets that respondents see, with the objective of obtaining a high-quality data set [7]. When creating the experimental design, there are several aspects that need to be taken into consideration, including (1) the analytical model specification, (2) whether the aim is to estimate main effects only or interaction effects as well, (3) whether the design is labeled or unlabeled, (4) the number of choice tasks and blocking options to be used, (5) which type of design of the choice matrix to use (eg, full factorial or fractional factorial, orthogonal or efficient), and (6) how the attribute-level balance will be achieved. These are now considered.

#### Analytical Model Specification

The first step in the generation of an experimental design is to specify the analytical model to estimate the parameters of the DCE. This step is an important component of choosing the type of choice matrix design, described later in this paper. The approach selected here needs to be accounted for when generating the structure of the experimental design.

A discrete choice model describes the probability that an individual will choose a specific alternative. This probability is expressed as a function of measured attribute levels specific to the alternative and of characteristics of the individual making the choice. This probability is represented by the dependent variable (the *choice variable*), which indicates the choice made by participants [8]. In this modeling framework, the attributes are the independent variables [8,13].

As part of the analytical model specification, knowing what type of statistical analysis will be used is key. Data analysis involves regression modeling in a random utility framework [8]. The random utility model conventionally used is also based on the Lancaster theory of consumer demand [35], which together assume that individuals make trade-offs when making a decision and would choose an option that offers the greatest utility [36], determined by how much importance they place on the attributes associated with the product [37].

The multinomial logit (MNL) model has been previously described as the “workhorse” of DCE estimation [38,39], and it typically serves as a starting point for basic model estimation (although alternative models, such as probit, may be used). It is important to note that MNL requires some important assumptions and limitations—for example, independence of irrelevant alternatives, homogeneity of preferences, and independence of observed choices [40,41]. Extensions of MNL (eg, nested logit, mixed logit, and latent class models) may be used to account for these limitations [39,40].

Based on the model specified in our DCE, the underlying utility function for alternative *j* [38] is shown in Textbox 1.

**The utility function used in our DCE research. DCE: discrete choice experiment.**

U_j_ = (β_cost_ × X_j__cost_) + (β_developer_ × X_j__developer_) + (β_ratings_ × X_j ratings_) + (β_description_ × X_j__description_) + (β_images_ × X_nj_ _images_) + ε

Note:

1) U is the overall utility derived from alternative j.

2) β is the coefficient attached to X_j_ estimated in the analysis and represents the part-worth utility attached to each attribute level.

3) ε is the random error of the model—in other words, the unmeasured factors influencing the variation of preferences.

#### Main Effects or Interaction Effects

The next step in model specification is deciding whether main effects or interaction effects will be investigated. The main effects, the most commonly used, investigate the effect of each attribute level on the choice variable. The effect on the choice variable gained by combining two or more attribute levels (eg, app developer and the app's monthly cost) refers to an interaction effect [13]. In our DCE, given the novel nature of the research on the uptake of health apps and the lack of empirical evidence to suggest the presence of potential interactions between attributes, we decided to only look at main effects.

#### Labeled or Unlabeled Experiment

In a labeled experiment, the alternatives are specific and different (eg, smartphone app-based smoking cessation intervention vs website-based smoking cessation intervention) and alternative specific attributes could be used (eg, some attributes relevant only for apps and others for websites). This is in contrast to an unlabeled experimental design, where the alternatives are unspecified (eg, smoking cessation app alternative 1 vs smoking cessation app alternative 2) and also must have the same attributes. Given that a DCE model estimates parameters for each of the alternatives being considered, these alternative specific parameters must be included in the structure of the experimental design (described in the next section) in a labeled experiment; in an unlabeled experiment, because alternative specific parameters are arbitrary, they are excluded [22,42,43]. In health economics, the unlabeled approach is the most common. In our DCE, the unlabeled approach was deemed logical here as we were comparing different presentations of the same app. Therefore, our DCE design applied an unlabeled approach.

#### Generation of the Structure of the Experimental Design

Once the model is specified, the structure of the experimental design can be generated. For this stage, hypothetical alternatives are generated and combined to form choice tasks, based on the chosen attributes and their levels. Several different software packages may be used to generate the experimental design of a DCE, such as Ngene, SAS, SPEED, SPSS, and Sawtooth. For our DCE, Ngene software was used [44].

##### Number of Choice Tasks and Blocking

The next step in the generation of an experimental design is to decide on the choice task and blocking. To minimize respondent and cognitive burden, and the risk of participants losing interest during the DCE task, consideration must be paid to the target population, the number of tasks, and their complexity [13]. The higher the number of attributes, alternatives, and choice tasks, the higher the task complexity [20]. The literature suggests that a feasible limit is 18 choice sets per participant [45,46]. In the review by Marshall et al [27], most studies included between 7 and 16 choice sets. In our DCE, we administered 12 choice tasks per participant, which were deemed a number low enough to avoid excessive cognitive load but high enough to establish sufficient statistical precision.

We developed 48 choice tasks and blocked them into 4 survey versions (12 choice tasks for each). Each block represented a separate survey, and participants were randomly assigned to one of the four survey versions. Blocking is a technique widely used in DCEs to reduce cognitive burden by partitioning large experimental designs into subsets of equal size, thereby reducing the number of choice tasks that any one respondent is required to complete [47]. Blocks were generated in Ngene software, which allows for the minimization of the average correlation between the versions and attributes’ levels [48]. For the blocking to be successful, the number of choice tasks included in one block must be divisible by the number of attribute levels; in our DCE, attributes had either three or four levels.

It is noteworthy that to undertake the sample size calculation, it is crucial to know the number of alternatives per choice set, the largest number of levels of any attribute (for DCEs looking at main effects only) or the largest level of any two attributes (for a DCE looking at interaction effects), and the number of blocks [38]. Therefore, DCEs using blocking require a larger sample size [47].

##### Type of Choice Matrix Design

Depending on the number of attributes and their levels, a full- or fractional-factorial design can be applied. A full-factorial design would include all possible combinations of the attributes’ levels and allow the estimation of all main effects and interaction effects independent of one another [20]. However, this type of design is often considered impractical due to the high number of choice tasks required [20]. To illustrate this, the formula of calculation of the possible unique choice alternatives for a full-factorial design is *L^A^*, where *L* represents the number of levels and *A* the number of attributes [39]. If the attributes in the DCE have a different number of levels, these need to be calculated separately and multiplied together. To reduce response burden, in our DCE, we generated a fractional-factorial design in Ngene [44], representing a sample of possible alternatives from the full-factorial design. This way, we were able to reduce the total 432 alternatives in the full design (given by *L^A^* = 4^2^ × 3^3^) to a fractional sample of 96 alternatives, arranged in 48 choice pairs.

Systematic approaches for generation of fractional-factorial designs may be further categorized into orthogonal design and efficient design. An orthogonal design is a column-based design based on orthogonal arrays that present properties of orthogonality (attributes are statistically independent of one another) and level balance (levels of attributes appear an equal number of times) and does not introduce correlation between the attributes [38]. An orthogonal array is an optimal design that is often used for DCEs examining main effects when the number of attributes and their levels is small.

For studies with five or more attributes with two or more levels, an orthogonal design may not be practical. There has therefore been a recent change in thinking toward a nonorthogonal and statistically more efficient design [38]. When perfect orthogonality and balance cannot be achieved or are not desirable, an efficient design can be applied [20]. In contrast to an orthogonal design, an efficient design aims to increase the precision of parameter estimates for a given sample size (ie, minimizing the standard errors of the estimated coefficients), while allowing some limited correlation between attributes. The most widely used efficiency measure is the D-error, which may be easily estimated using various software packages, such as Ngene, and refers to the efficiency of the experimental design in extracting information from respondents [21]*.* Experimental designs generated using this approach are known as D-efficient designs. A D-efficient experimental design is also recommended to maximize statistical efficiency and minimize the variability of parameter estimates [7].

An efficient design requires that known prior information about the parameters (known as priors) be made available to the algorithm and also requires the analyst to specify the analytical model specification, as described previously. Depending on what information is available, one of three types of D-efficient design can be generated [21]:

1. *D_z_-efficient* design (*z* stands for zero priors): If no prior information about the magnitude or directions of the parameters is available. D_z_-efficient design is an orthogonal design. This design assumes the parameters are zero.
2. *D_p_-efficient* design (*p* stands for priors): This assumes a fixed, certain value and direction for the parameters.
3. *D_b_-efficient* design (*b* stands for Bayesian): A Bayesian approach is whereby the parameter is not known with certainty but may be described by its probability distribution.

The best practice is to pilot the DCE. For the pilot phase, there is limited information available and using the D_z_-efficient or D_p_-efficient design is sensible. In our DCE, we chose to apply a D_p_-efficient design, as the direction of priors of the app was known from the previously conducted survey, to narrow down the attribute levels and to provide prior estimates of the parameters for the attribute levels. For example, we knew that a trusted organization will likely positively influence uptake and cost estimated negatively so. The direction of priors was assumed to be a small near-zero negative or a positive value for the design.

The pilot phase provided the estimation that we used to generate a D_b_-efficient design for the final DCE. It is noteworthy that when the parameter priors are different from zero, the efficient design generated produces smaller prediction errors than orthogonal designs [21,49,50]. Hence, a D-efficient design will outperform an orthogonal design, and (given reliable priors), a D_p_-efficient design will outperform a D_z_-efficient design [21]. Further, when reasonable assumptions about the distributions are made, a D_b_-efficient design will outperform a D_p_-efficient design. Therefore, it may be advisable to start piloting with a D_p_-efficient design and to generate a D_b_-efficient design for the final DCE. The DCE literature provides a detailed and more comprehensive description of orthogonal and efficient designs [21] and the approximation of the Bayesian efficient design [23].

##### Attribute-Level Balance in the Model

The attribute-level balance aims to ensure all attribute levels ideally appear an equal number of times in the experimental design. The allocation of the attribute levels within the experimental design can affect statistical power; if a certain level is underrepresented in the choice sets generated, then the coefficient for that level cannot be easily estimated. How attributes levels are distributed is therefore an important consideration when designing the choice sets. Dominant alternatives, where all attribute levels of one alternative are more desirable than all attribute levels in the others, do not provide information about how trade-offs are made, as individuals usually would select the dominant alternatives. Therefore, avoiding dominant alternatives in the experimental design is important and can be achieved by consulting the software manual to ensure the correct algorithm is used. The syntax used in Ngene to generate choice sets of the pilot phase and more information about the algorithm used can be accessed on the Open Science Framework [19].

#### Piloting the DCE and Generating the Bayesian Design

In addition to providing estimations for the choice matrix design described above, piloting offers an opportunity to ensure that the information is presented clearly and that the choices are realistic and meaningful. It also provides insight into how cognitively demanding it is for respondents to complete. This can be achieved by gathering feedback on the survey completion process. The findings of the pilot may suggest that the DCE needs to be amended, such as reducing the number of choice sets or the number of attributes, so that the responses are a better reflection of the participants’ preferences and improve the precision in the parameter estimates [13].

There is no formal guidance on how large the pilot sample should be, and this is largely guided by the budget and complexity of the experimental design. Accuracy of the priors will improve with increasing sample size, but as few as 30 responses may be sufficient to generate useable data [44]. In our pilot study conducted with 49 individuals, feedback from the participants suggested that with the initial order of the attributes, there was a tendency to ignore the last two attributes, app description and images of the app, the most text-heavy attributes. This may have compromised the examination of the relative importance of those two attributes (app description and images of the app). Therefore, we decided to change the final order of the attributes from (1) *monthly price of the app*, (2) *the ratings of the app*, (3) *who developed the app*, (4) *the description*, and (5) *images shown* to the one listed in Figures 1 and 2. The longest completion time for the survey was under 12 min. Thus, we concluded that the number of choice tasks did not need to be reduced.

In our research, the data from the pilot phase were analyzed using the freely available Apollo package in R software [51]. The coefficients and their standard errors from the output were used as priors to generate the final choice sets using the Bayesian efficient design following the steps described previously. The syntax used in R used to analyze the pilot data and that used to generate the Bayesian efficient design in Ngene can be accessed on the Open Science Framework [19].

### Internal Validity

Assessing the internal validity of a DCE can help with understanding the consistency and trade-off assumptions made by participants [52]. There are several ways to examine the internal validity of a DCE. For example, in the *stability validity test,* a choice task would be repeated later in the sequence to investigate the consistency of the participants’ decision, whether they would choose the same alternative [52]. Another way to test internal validity is the *within-set dominated pairs* type of internal validity, in which one alternative is a dominant alternative in which all attributes are the most desirable ones. The choice sets designed to measure internal validity are excluded from the analysis. There are several internal validity tests that are built into software packages such as MATLAB [52], although these can be produced manually as well. In our research, we used the stability validity test to check the internal validity by repeating a randomly generated choice task (in our case, it was the fourth). Therefore, participants were shown 12 choice tasks, plus an additional hold-out task. The data from the randomly generated hold-out task were excluded from the analysis.

Although internal validity checks provide some measure of data quality, it should be noted that answering a repeat choice inconsistently is not a violation of random utility theory [53]. Furthermore, there is no consensus on what to do with the data from responses that fail validity tests. Following the advice of Lancsar and Louviere [54], we did not exclude participants who failed the internal validity check, as that might have caused statistical bias or affected statistical efficiency. However, we reported data on internal validity to enable the reader to make a judgement on likely biases.

All additional study materials used in our example, including the full data set and the results of the DCE, can be accessed on Open Science Framework [19].

## Discussion

### Summary

This paper describes the development of a DCE, following the stages required to establish attributes and their levels, construct choice tasks, define the utility model, decide on labeled and unlabeled choices to apply, decide on the number of choice tasks that need to be generated, and make decisions on the structure of the experimental design, how to achieve attribute-level balance, how to assess the internal model validity, and how to pilot-test. In doing so, the intention is to advance methodological awareness of the application of stated preference methods in the field of digital health, as well as to provide researchers with an overview of their application using a case study of a DCE of smoking cessation app uptake.

Although DCEs are widely used to understand patient and provider choices in health care [8,10,15,55], they have only recently started to gain popularity in digital health [4-6] and as such represent an underused approach in digital health. With the growing evidence of the benefit of digital health initiatives, there are clear benefits to widening the application of DCEs so that they may more routinely inform digital health development, inform digital tool presentation, and, most importantly, predict uptake and engagement with digital products. Although several attempts have been made to measure engagement with digital tools using a wide range of methodologies [56-58], the insights we have from them that can be translated to uptake are limited. One plausible explanation is that uptake of digital tools is difficult to empirically measure.

### Benefits and Limitations of DCEs

DCEs bring several benefits to help overcome the issue of measuring uptake in digital health or in other areas where the measurement of the predictors of uptake in a good or service is required. For example, as illustrated by the case study here, they enable the researcher to gain measurable insights into situations in which quantitative measures are hard to otherwise obtain, such as the factors impacting the uptake of health apps on curated health app portals. A DCE also helps to quantify preferences to support more complex decisions [59]. An example would be the consideration of how to plan the development of an app that would provide appealing looks or features that would promote uptake. The DCE methodology is also considered a convenient approach to investigate the uptake of new interventions, including digital health interventions [38], for example, digital behavior change interventions using a health and well-being smartphone app. Therefore, DCEs can be used in hypothetical circumstances, enabling the measurement of preferences for a potential policy change or digital health system change before it is implemented [13], such as the recent investigation of the uptake of a COVID-19 test-and-trace health app [3,4]. The experimental nature of the DCE also means that participants’ preferences can be recorded based on controlled experimental conditions, where attributes are systematically varied by researchers to obtain insight into the marginal effect of attribute changes on individuals’ choices [7].

Despite their benefits, the application of DCEs presents several challenges. As with all expressed preference methodologies, the hypothetical nature of the DCE choice set raises concerns about external validity and the degree to which real-world decisions might equate to those made by study participants under experimental conditions, a phenomenon known as the intention-behavior gap [60]. As such, participants may believe they would choose a scenario presented and described in a choice task, but in real life, there might be other factors that would influence their behaviors, such as the aesthetics of the app [28]. This limitation can at least partially be overcome by developing convincing and visually appealing choice tasks. Nevertheless, to date, there has been limited progress in testing for external validity due to the difficulty in investigating preferences in the real world [38]. Indeed, a recent systematic review of the literature on DCEs in health care reported that only 2% of the included studies (k=7) report details of the investigation of external validity [47], while an earlier systematic review and meta-analysis (k=6) found that DCEs have only a moderate level of accuracy in predicting behaviors of health choices [61]. To our knowledge, no study has been published that investigates the external validity of a DCE developed in digital health. One potential opportunity to undertake some testing would be through a curated health app portal, where the same health app is presented in two or more different ways. With the help of website analytics, actual user behavior could be measured in this situation.

A final significant concern associated with the use of a DCE is that any single choice set is unlikely to be able to present the user with all relevant attributes, regardless of how well it has been developed [61]. Choosing the most relevant attributes to test in a DCE, therefore, requires comprehensive preparatory research, which can lengthen the time required to undertake the development phase of any piece of work.

### Conclusion

In summary, DCEs have significant potential in digital health research and can serve as an important decision-making tool in a field where observational data are lacking. We hope that the content of this paper provides a useful introduction and guide to those interested in developing such experiments in digital health.

## Abbreviations

DCE: discrete choice experiment
MNL: multinomial logit
